# Our Brethren Across the Line

**Published:** 1887-10

**Authors:** 


### OUR BRETHREN ACROSS THE LINE.

A prominent dentist of Ontario writes us that, so far as regarded the organization of the Congress, "the dental profession of Ontario has been absolutely ignored." In answering his letter, may we not, without offense, say a word to our brethren of the Upper Province, for the remarks we desire to make do not apply to the Lower Province of Quebec.

If the dentists of Ontario were not consulted about the organization of the Dental Section of the Congress, was there not a reason for it? Where were the authorities to find a man who has sufficiently identified himself with society work to warrant placing him upon the list of officers? The dentists of the Province of Quebec were recognized by nominating one of their number as a Vice-President, although he did not attend the meeting. But Ontario, with its five hundred dentists, is without a dental society, without a literature of its own, and practically without membership in any of our societies. As often as every third year the American Dental Association has met upon the border line, as near to Canada as it could possibly get, but not one member of the profession from Ontario has joined it, and very few indeed have shown themselves, for even a single session, at its meeings. Yet there is no reason why they may not become active members. How many in Ontario show any interest in American professional matters? Probably they need, and can be benefited by society meetings as much as we can be, and if this be not the case, there is most assuredly a crying necessity for their presence on our own account. Professional lines should not follow territorial boundaries, for science knows no bourn, no State limit.

Ontario has no professional journal of its own, and when one was established by men who desired to be progressive, despite chilly surroundings, Dr. Chittenden, of Hamilton, was left to carry the Ontario end alone, for it met with no support worthy the name.

The impression in the United States is too general, that for some reason the Ontario dentists do not desire any close relationship with us ; that when the law was enacted it was so framed as to place in the hands of a few the control of professional matters in Ontario ; that it was desired to withdraw Ontario dentists from the influence of the profession here ; that the administration of affairs was so conducted as to discourage attendance upon American colleges, to the end that students should be forced into their own school—a perfectly legitimate action so long as the motives which prompted it were unselfish, but a course which tended to bring about an isolation.

It takes time and labor, aye, and money, to keep up societies and to support a respectable literature, and we on this side the great lakes devote considerable of each to the work, because we have a genuine love for our profession, and are not alone solicitous about what it shall bring us. As regards the late Congress, few can appreciate the amount of time and money that were freely devoted to it. But how much of either does any one imagine was received from Ontario ? Not a moment of the one or a cent of the other, so far as we know. Invitations would have been issued to the leading dentists, had not the necessity for them been forestalled by the action of the American Medical Association, but there was not a surface indication that such solicitations to attend would be accepted, or even be graciously received. Not that any one imagines Canadians to be boors, who were capable of receiving such invitations discourteously, but they had shown no sign of any interest in the matter.

Americans cannot be accused of a spirit of exclusiveness, for at the invitation of a single member from the Province of Quebec, one of our most important societies held its regular meeting in Montreal this summer, where the members were most royally entertained, and even now they are sighing for another chance to go. When has Ontario exhibited a like professional spirit ? It is those who show the possession of a friendly feeling that have many friends.

Let Ontario dentists exhibit a true professional spirit ; let them actively engage in general professional work ; let them show their interest in society matters by sustaining a society of their own, or attending in large numbers the meetings upon this side ; let them indicate a genuine love for and interest in professional matters aside from the bread-and-butter aspect ; let them mount dentistry as a Pegasus, and not ride it like a cart-horse, and our word for it they will often enough be called upon to share the counsels of the men who are actively engaged in general professional work.

We hope that no one will for a moment imagine that this is written in a carping or captious spirit. Some of our dearest friends live across the border, and no one better knows the signal ability, the real generosity and disinterestedness which exist beneath the crust of conventionality that veils the hearts of very many Ontario dentists. It is because of this knowledge, and because we know that they are not exhibiting themselves in the best light, because we wish to meet them more often, and because they are aware of our real regard for them, living so near them as we do, that we have ventured upon the endeavor to stir them up, and if the pole has a sharp point, perhaps it will be none the less effectual.

